# Phantom validation of 4D flow: independent validation of flow velocity quantification using particle imaging velocimetry

**DOI:** 10.1186/1532-429X-17-S1-O18

**Published:** 2015-02-03

**Authors:** Johannes Töger, Sebastian L Bidhult, Johan Revstedt, Marcus Carlsson, Håkan Arheden, Einar Heiberg

**Affiliations:** 1 Cardiac MR group Lund, Dept. of Clinical Physiology, Lund University, Lund, Sweden; 2 Numerical Analysis, Centre for Mathematical Sciences, Lund University, Lund, Sweden; 3 Department of Biomedical Engineering, Faculty of Engineering, Lund University, Lund, Sweden; 4 Department of Energy Sciences, Lund University, Lund, Sweden

## Background

4D phase contrast magnetic resonance blood flow measurements (4D PC-MR) is a promising tool for clinical assessment of cardiac function, but lack of independent validation may limit clinical adoption. Previous validation studies are limited by lack of pulsatility [1,2] or by the need of a post hoc correction factor to compensate for differences in experimental setup between 4D PC-MR and reference imaging [3]. Therefore, we aimed to validate 4D PC-MR velocity measurements with particle imaging velocimetry (PIV) as the reference standard in a phantom setup based on vortex ring formation, which has previously shown excellent repeatability [4].

## Methods

**Figure 1:**
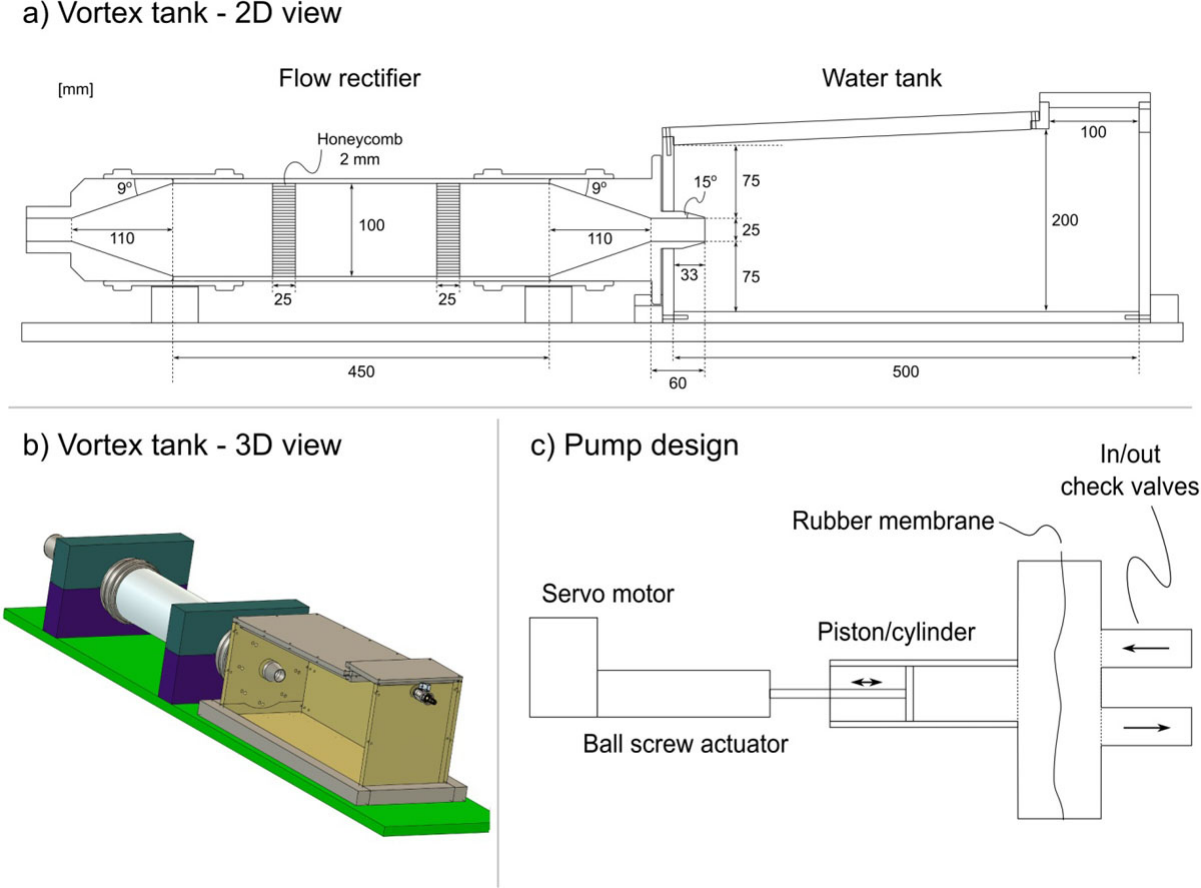
**Design and construction of the vortex ring flow phantom.** a) 2D view of the flow rectifier and vortex ring tank. All measures are in millimeters (mm). b) 3D view of the flow rectifier and vortex ring tank. c) Pump design. A servo motor powers a ball screw linear actuator, which in turn moves a piston-cylinder apparatus. A rubber membrane separates the flow medium from the pump to prevent abrasive particles from entering the piston/cylinder apparatus.

We constructed a pulsatile pump and a water tank with a 25mm nozzle (Figure 1). Five different pump settings were used. PIV measurements with spatial resolution 1.5x1.5 mm were obtained on two consecutive days to evaluate phantom stability. Nozzle stroke volumes were measured using 2D PC-MR (voxel size 2.4x2.4x6 mm, VENC 50 cm/s). 4D PC-MR (3x3x3 mm voxels, temporal resolution 50 ms, VENC 100 cm/s) was acquired at 1.5T using a 32-channel cardiac coil and SENSE=2. The 4D PC-MR velocity measurements were interpolated to the PIV resolution, and compared to PIV pixel-by-pixel after semi-automatic registration.

## Results

**Figure 2:**
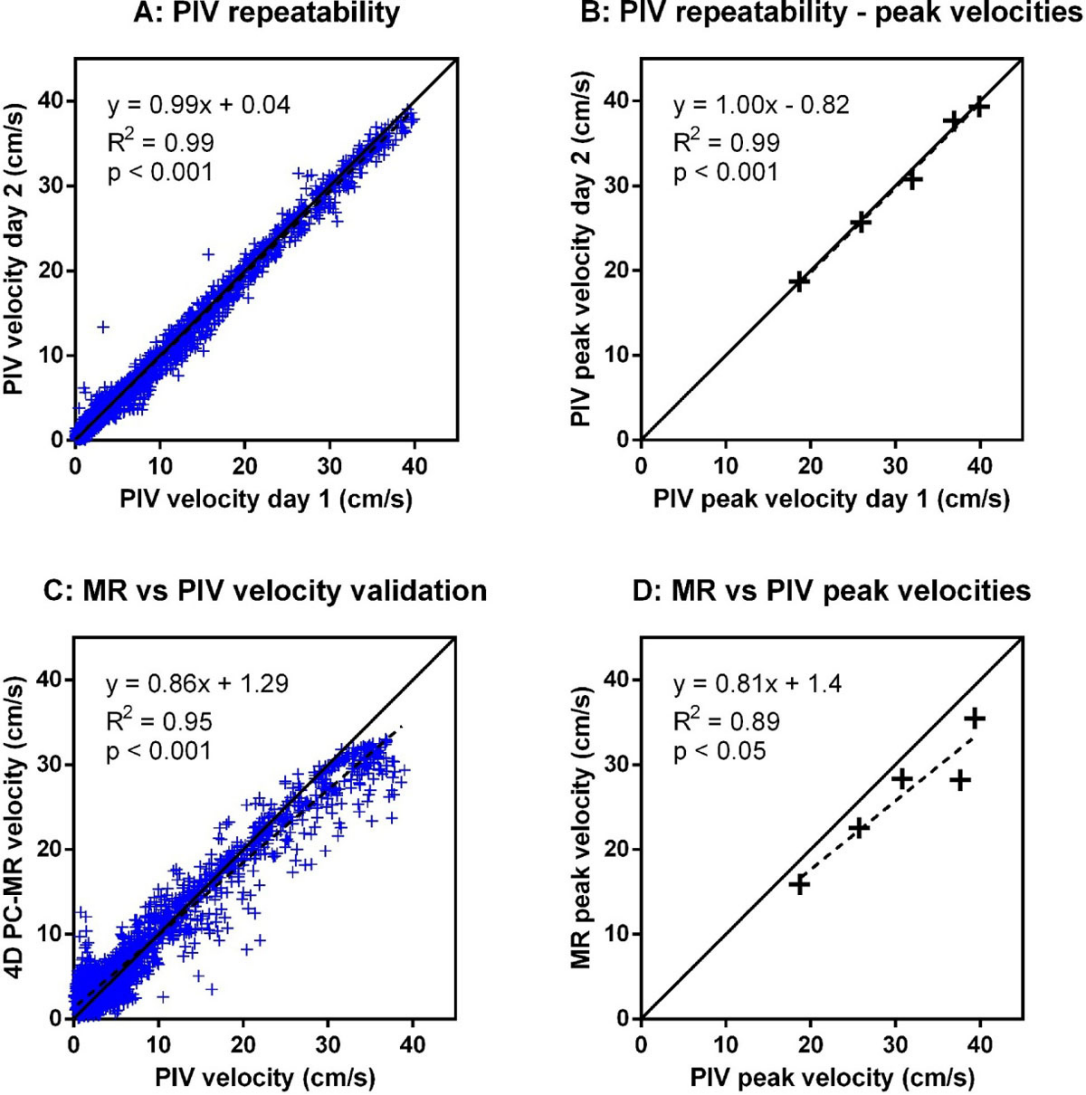
**Experimental results.** Panel A: Pixel-wise PIV repeatability for the highest pump setting. Panel B: PIV repeatability for peak velocities for all five pump settings. Panel C: Validation of 4D PC-MR velocities against PIV for the highest pump setting. Panel D: Validation of 4D PC-MR peak velocities against PIV for all five pump settings. 4D PC-MR underestimated peak velocities by 8-25% compared to PIV. All data taken at 400 ms from the initiation of flow.

Stroke volumes ranged from 12-37 ml and peak nozzle velocities from 19-40 cm/s. PIV velocities showed excellent agreement between days, both for the pixel-wise comparison (Figure 2A, R^2^=0.99, bias -0.04±0.72 cm/s) and for peak velocities (Figure 2B, R^2^=0.99, bias -1±2%). Validation of 4D PC-MR against PIV showed good pixel-wise agreement (Figure 2C, R^2^ = 0.95, bias 0.17±2.31 cm/s), but peak velocities were underestimated by 8-25% (Figure 2D, R^2^=0.89, bias -14±7%).

## Conclusions

This study shows that 1) the proposed vortex ring flow phantom can be used for validation of 4D PC-MR measurements, 2) 4D PC-MR shows good overall agreement in velocity for the pixel-wise comparison, but underestimates peak velocities by 8-25% compared to PIV.

## Funding

This study was supported by Swedish Research Council grants VR 621-2005-3129, VR 621-2008-2949, and VR K2009-65X-14599-07-3, National Visualization Program and Knowledge Foundation grant 2009-0080, the Medical Faculty at Lund University, Sweden, the Region of Scania, Sweden and the Swedish Heart-Lung Foundation.
